# The hidden blood loss and its factors in patients undergoing minimally invasive knee arthroscopy

**DOI:** 10.3389/fsurg.2022.944481

**Published:** 2022-08-30

**Authors:** Sheng Li, Liang A

**Affiliations:** ^1^ Department of Orthopaedics, Affiliated Central Hospital of Shenyang Medical College, Shenyang, China; ^2^ Shenyang Hand Foot Clinical Research Center, Shenyang, China

**Keywords:** arthroscopy, minimally invasive, knee, hidden blood loss, risk factors

## Abstract

**Background:**

With respect to knee arthroscopy, assessing the amount of hidden blood loss is of great importance to avoid potential complications such as fever, anemia, difficulty in wound healing and wound infection. The current study aims to investigate the hidden blood loss and its factors in patients who underwent minimally invasive knee arthroscopy.

**Methods:**

Consecutive patients with knee joint injury, who underwent arthroscopic minimally invasive treatment, were enrolled from January 2019 to November 2020 and were retrospectively studied. Demographic information on these patients, such as medical history and biochemical parameters, was collected. The hidden blood loss was calculated. Multivariate linear regression analysis was used to confirm independent factors associated with hidden blood loss.

**Results:**

Finally, a total of 100 patients aged 44.78 ± 13.67 (range 17–66) years were reviewed, and it was found that a substantial amount of [387.02 ± 252.56 (range 18.89–1130.06) ml] hidden blood loss occurred after minimally invasive knee arthroscopy. Univariate analysis showed that this hidden blood loss was negatively correlated with age, gender, postoperative hemoglobin, and postoperative hematocrit (all *P* < 0.05), while it was positively correlated with body mass index (BMI), length of hospital stay, preoperative red blood cells, preoperative hemoglobin, preoperative hematocrit, blood volume, and the presence of medical conditions (all *P* < 0.05). Further multivariate linear regression indicated that preoperative hematocrit, blood volume, and postoperative hematocrit were independent factors associated with actual blood loss, and preoperative hematocrit, blood volume, postoperative hematocrit, and gender were independent factors associated with hidden blood loss, respectively (all *P* < 0.05).

**Conclusion:**

Preoperative hematocrit, preoperative blood volume, postoperative hematocrit, and gender are the influencing factors of hidden blood loss in patients undergoing minimally invasive treatment under knee arthroscopy. More attention should be paid to hidden blood loss and its factors during the perioperative period.

## Introduction

Arthroscopy provides a technical guarantee for the minimally invasive treatment of orthopedic knee joint injury. Compared with traditional open surgery, it has the advantages of minimized surgical trauma, less bleeding, and accelerated postoperative rehabilitation (1). At present, it is being widely used in medical surgery for sports orthopedics and is achieving good clinical results (2). However, the relevant complications after arthroscopy are also becoming a matter of wide concern, and a few examples of these are fever, anemia, difficulty in wound healing, and wound infection. In response to these complications, clinicians have found many causes, of which hidden blood loss after knee arthroscopy is being paid increasing attention by everyone, following which it has become a non-negligible factor (3). Hidden blood loss is not usually mentioned during general assessment because of its invisibility, while an association has been found between increased blood loss and perioperative complications (4). Concerning minimally invasive knee arthroscopy, evaluating hidden blood loss is of great importance to prevent the aforementioned potential complications.

Since researchers proposed the concept of hidden blood loss in 2000 (5), a series of studies on various treatments for this blood loss in the field of orthopedic surgery has been reported successively, such as minimally invasive treatment of tibial fractures (6), minimally invasive treatment of fractures, knee replacement, hip replacement, and spinal surgery (7). To the best of our knowledge, there was no report in the literature on the factors causing hidden blood loss from minimally invasive treatment with knee arthroscopy. Herein, the current study retrospectively reviewed the medical data of patients who underwent minimally invasive knee arthroscopy to evaluate hidden blood loss and identify its associated factors.

## Patients and methods

The study protocol was approved by the Institutional Review Committee (IRB) of the Central Hospital Affiliated with Shenyang Medical College and followed the tenets of the Declaration of Helsinki. The IRB confirmed that informed consent was waived due to the retrospective nature of the study design and data were analyzed anonymously. From January 2019 to November 2020, 100 consecutive patients who underwent arthroscopically minimally invasive treatment for knee injuries in our hospital met our inclusion criteria and were enrolled in our study. The inclusion criteria were as follows: (1) fresh knee joint injury in one limb, no other serious trauma; (2) minimally invasive arthroscopic knee treatment; (3) blood parameters before surgery and within 3 days after surgery; (4) no hematological diseases (hemophilia or vitamin K deficiency, etc.); and (5) no history of anticoagulant drugs. The exclusion criteria were as follows: patients (1) younger than 16 years; (2) those with previous knee surgery; (3) knee surgery combined with serious blood-related diseases; and (4) oral anticoagulants before 3 months. Demographic information such as age, gender, medical history, diagnosis, and laboratory investigations as well as pre-, intra-, and postoperative data were collected *via* the electronic medical record system.

### Surgical procedure invasive operation

An incision of approximately 0.5 cm in the anterolateral and medial sides of the knee was performed, the arthroscopy was located to check the joint cavity, and then the planer was cleaned to determine the injury of the cruciate ligament and meniscus. The hamstring tendon was taken out as the transplanted tendon. The edge of the irregular meniscus was removed and the torn meniscus was sutured. The process of making the cruciate ligament bone tunnel included (1) locating with a femoral and tibial guide; (2) drilling into the Kirschner wire; (3) drilling through the bone cortex with a hollow drill; (4) measuring the bone tunnel; and (5) selecting an appropriate hollow drill to drill an appropriate depth according to the tendon thickness.

### Calculation of hidden blood loss

Hidden blood loss (ml) = actual total blood loss − overt blood loss + transfusion volume;

Actual total blood loss (ml) = blood volume × (preoperative hematocrit − postoperative hematocrit)/mean hematocrit (Gross equation)

The estimated blood volume (EBV) of each patient was calculated by using the Nadler formula (8). EBV (L) = *k*_1_ × height (m)^3^ + *k*_2_ × weight (kg) + *k*_3_, where *k*_1_ = 0.3669, *k*_2_ = 0.03219, and *k*_3_ = 0.6041 for males and *k*_1_ = 0.3561, *k*_2_ = 0.03308, and *k*_3_ = 0.1833 for females.

### Statistical analysis

SPSS v20.0 for Windows (IBM Corp., Armonk, NY, USA) was used for data analysis. The Shapiro–Wilk test was used to assess the normality of data distribution. Continuous data were described as either mean ± standard deviation (SD) or median and range based on their distribution. A comparison among different categories was assessed by using the Student's *t-*test. Pearson's correlation analysis and Spearman's correlation analysis were performed according to the normality of data distribution. Multiple linear regression was conducted for evaluated associated factors on blood loss. A *P*-value of less than 0.05 was considered statistically significant.

## Results

All demographic and baseline characteristics of the included participants are summarized in Table 1. Among 100 patients, there were 58 (58%) males and 42 (42%) females, aged 17–66 years, with a mean of 44.78 ± 13.67 years. Their mean BMI was 18.59–36.98 kg/m^2^. The mean total actual blood loss was 512.32 ± 317.29 ml, and the mean hidden blood loss was 387.02 ± 252.56 ml.

**Table 1:** Overall characteristics of the study population.

Variables	Mean ± SD (range)
Male/female	58/42
Age (years)	44.78 ± 13.67 (17–66)
BMI (kg/m^2^)	25.07 ± 3.67 (18.59–36.98)
Operation time (min)	114.9 ± 47.08 (48–270)
Hospital stay (days)	12.86 ± 10.16 (4–90)
Preoperative RBC [10(12)/L]	4.86 ± 0.53 (3.7–6.15)
Preoperative hemoglobin (g/L)	148.45 ± 15.57 (99–186)
Preoperative hematocrit (%)	43.43 ± 4.37 (31.1–53.2)
Postoperative RBCl [10(12)/L]	4.3 ± 0.52 (2.78–5.55)
Postoperative hemoglobin (g/L)	131.58 ± 16.2 (77–172)
Postoperative hematocrit (%)	38.35 ± 4.42 (24.2–49)
Blood volume (L)	4.48 ± 0.68 (3.2–6.28)
Actual total blood loss (ml)	512.32 ± 317.29 (18.89–1380.06)
Hidden blood loss (ml)	387.02 ± 252.56 (18.89–1130.06)

SD, standard deviation; BMI, body mass index; RBC, red blood cell.

In terms of total actual blood loss, male patients (*P* = 0.001) without previous medical disease (*P* < 0.001) had a higher total actual blood loss postoperatively. Similar results were found in hidden blood loss assessments (Table 2).

**Table 2:** Characteristics of included patients.

	Total actual blood loss	*P*	Hidden blood loss	*P*
Gender				
Male (*n* = 58)	595.30 ± 348.76	0.001	446.52 ± 284.4	0.003
Female (*n* = 42)	397.72 ± 225.39		304.86 ± 172.21	
Age				
≥50 (*n* = 53)	454.95 ± 293.89	0.08	344.1 ± 231.15	0.11
<50 (*n* = 47)	563.19 ± 331.12		425.08 ± 266.53	
BMI				
≥27 (*n* = 25)	570.71 ± 295.01	0.27	428.31 ± 233.98	0.35
<27 (*n* = 75)	492.86 ± 323.93		373.26 ± 258.48	
Medical disease or not				
Yes (*n* = 5)	193.38 ± 108.63	<0.001	143.38 ± 73.89	<0.001
No (*n* = 95)	529.12 ± 315.97		399.84 ± 252.24	
Epilepsy				
Right (*n* = 46)	485.82 ± 306.62	0.44	366.9 ± 247.99	0.46
Left (*n* = 54)	534.89 ± 327.27		404.16 ± 257.45	
Mechanism of injury				
Low (*n* = 12)	478.82 ± 375.5	0.69	373.82 ± 305.91	0.85
High (*n* = 88)	516.89 ± 310.74		388.82 ± 246.43	
Surgical method				
Single (*n* = 34)	460.2 ± 312.81	0.24	349.32 ± 247.06	0.29
Multiple (*n* = 66)	539.17 ± 318.61		406.44 ± 255.03	
Early postoperative complications				
Yes (*n* = 35)	508.11 ± 307.48	0.92	382.12 ± 243.21	0.89
No (*n* = 65)	514.58 ± 324.8		389.66 ± 259.28	

BMI, body mass index.

In correlation analysis, age, gender, postoperative hemoglobin, postoperative hematocrit, BMI, length of hospital stay, preoperative red blood cells, preoperative hemoglobin, preoperative hematocrit, blood volume, and the presence of medical conditions were correlated with actual blood loss and hidden blood loss, respectively (all *P* < 0.05, Table 3).

**Table 3:** Correlation between various indicators and actual blood loss and hidden blood loss.

	Actual blood loss		Hidden blood loss	
	*R*	*P*	*R*	*P*
Age (years)	−0.28	**<0.001**	−027	**0.007**
BMI (kg/m^2^)	0.22	**0.022**	0.20	**0.046**
Operation time (min)	0.15	0.13	0.15	0.15
Hospital stay (days)	0.24	**0.02**	0.25	**0.01**
Preoperative RBC [10(12)/L]	0.45	**<0.001**	0.43	**<0.001**
Preoperative hemoglobin (g/L)	0.38	**<0.001**	0.37	**<0.001**
Preoperative hematocrit (%)	0.43	**<0.001**	0.42	**<0.001**
Postoperative RBCl [10(12)/L]	−0.13	0.21	−0.15	0.15
Postoperative hemoglobin (g/L)	−0.19	**0.05**	−0.21	**0.03**
Postoperative hematocrit (%)	−0.22	**0.03**	−0.23	**0.02**
Blood volume (L)	0.37	**<0.001**	0.35	**<0.001**
Medical disease or not	0.27	**0.007**	0.28	**0.005**
Epilepsy	0.09	0.39	0.09	0.37
Mechanism of injury	0.07	0.51	0.05	0.61
Surgical method	0.12	0.22	0.12	0.24
Early postoperative complications	0.008	0.94	0.012	0.91
Gender	−0.27	**0.006**	−0.24	**0.02**

BMI, body mass index; RBC, red blood cell.

Multivariate linear regression analysis demonstrated that preoperative hematocrit (*β* = 100.407, *P* < 0.001), blood volume (*β* = 134.497, *P* < 0.001), and postoperative hematocrit (*β* = −90.944, *P* < 0.001) were independently associated with actual blood loss (Table 4). Furthermore, preoperative hematocrit (*β* = 78.613, *P* < 0.001), blood volume (*β* = 119.371, *P* < 0.001), postoperative hematocrit (*β* = −72.719, *P* < 0.001), and gender (*β* = 52.280, *P* = 0.011) were independent factors for hidden blood loss (Table 5).

**Table 4:** Multiple regression analysis related to actual blood loss.

	*B*	95% CI		*P*
		Lower	Upper	
Age (years)	−0.232	−1.057	0.592	0.577
BMI (kg/m^2^)	−1.790	−5.373	1.793	0.323
Hospital stay (days)	−0.035	−1.013	0.943	0.944
Preoperative RBC [10(12)/L]	−39.513	−87.933	8.908	0.108
Preoperative hemoglobin (g/L)	0.812	−1.815	3.440	0.540
Preoperative hematocrit (%)	**100.407**	**90.252**	**110.562**	**<0.001**
Blood volume (L)	**134.497**	**105.895**	**163.100**	**<0.001**
Postoperative hemoglobin (g/L)	−2.350	−5.626	0.926	0.158
Postoperative hematocrit (%)	**−90.944**	**−102.434**	**−79.453**	**<0.001**
Medical disease or not	−10.955	−53.457	31.546	0.610
Gender	26.666	−10.031	63.362	0.152

BMI, body mass index; RBC, red blood cell; CI, confidence interval.

Bold values indicate data are statistically significant.

**Table 5:** Multiple regression analysis associated with hidden blood loss.

	*B*	95% CI		*P*
		Lower	Upper	
Age (years)	0.018	−0.875	0.911	0.967
BMI (kg/m^2^)	−3.722	−7.603	0.160	0.060
Hospital stay (days)	0.277	−0.782	1.337	0.604
Preoperative RBC [10(12)/L]	−29.964	−82.413	22.486	0.259
Preoperative hemoglobin (g/L)	1.083	−1.763	3.928	0.452
Preoperative hematocrit (%)	**78.613**	**67.613**	**89.614**	**<0.001**
Blood volume (L)	**119.371**	**88.388**	**150.354**	**<0.001**
Postoperative hemoglobin (g/L)	−1.845	−5.394	1.704	0.304
Postoperative hematocrit (%)	**−72.719**	**−85.166**	**−60.273**	**<0.001**
Medical disease or not	−21.102	−67.140	24.936	0.365
Gender	**52.280**	**12.530**	**92.030**	**0.011**

BMI, body mass index; RBC, red blood cell; CI, confidence interval.

Bold values indicate data are statistically significant.

## Discussion

To date, the most common complications occurring after arthroscopic surgery may be related to hidden blood loss, which is to rule out significant blood loss during surgery and postoperative drainage volume, as the part of the body’s hidden loss of blood volume. Hidden blood loss is generally associated with hematocele in the joint space, interstitial hematocele, hemolysis, and stress gastrointestinal bleeding leading to hemoglobin loss (9). However, there are few reports on the factors behind hidden blood loss after knee arthroscopic surgery. To address this research gap, our study demonstrated that preoperative hematocrit, blood volume, and postoperative hematocrit were independent factors associated with actual blood loss, and preoperative hematocrit, blood volume, postoperative hematocrit, and gender were independent factors associated with hidden blood loss, respectively.

Numerous studies have shown that hidden blood loss is also an important factor in other surgeries that also belong to the minimally invasive category in clinical practice. Femoral fractures are common in the elderly, with more than 10% of European fracture patients dying within 30 days and more than 25% within 1 year (10). Anemia is one of the important factors associated with mortality, which increases the incidence of lung and brain diseases and disrupts the balance of oxygen supply. At present, the intramedullary nailing fracture technique used in clinical practice is a relatively minimally invasive surgery, which reduces the surgical incision of patients and also the intraoperative visible blood loss on the surface. However, patients still show symptoms of anemia in the perioperative period, which is inseparable from hidden blood loss. Hidden blood loss accounts for a high proportion of total perioperative blood loss in patients with femoral fractures. If its influence is not considered, it often leads to anemia or hypovolemia, which will affect the postoperative recovery of patients (11). If the perioperative blood loss of elderly patients with intertrochanteric fractures is 863.8 ± 429.9 ml, then the hidden blood loss will be 772.3 ± 424.7 ml (12), and so it can be seen that the hidden blood loss is predominant. This is consistent with the results obtained by researchers (4), who observed that the total blood loss of more than 500 patients with fractures was approximately 6 times the operative blood loss. Intramedullary nailing is also used by surgeons in minimally invasive surgery for tibial fractures because of its wide indications, minimal trauma, reliable fixation, and antirotation. Also, due to less intraoperative blood loss, total blood loss in the perioperative period of patients is rarely noticed. However, when reaming intramedullary nails are used to fix tibial fractures, intramedullary nails damage the medulla and significantly increase hidden blood loss (13). Endoscopic lumbar discectomy has been standardized as a typical minimally invasive surgical technique for lumbar disc herniation (14), and studies have shown that this minimally invasive surgery also presents with a large amount of hidden blood loss (15).

The mechanism of hidden blood loss can be summarized as blood stasis in the joint cavity and interstitial space, hemolysis, and gastrointestinal bleeding. Even if wound drainage is done after surgery, bleeding in the patient’s surgical area cannot be completely discharged, and some bleeding remains in the joint cavity and tissue space and is gradually absorbed, organized, and exuded. These hemorrhages are large and do not participate in the systemic circulation, inducing the loss of hemoglobin. This may be related to the abnormal coagulation mechanism of patients suffering from rheumatoid arthritis (16) and blood system bleeding diseases. Perioperative use of anticoagulant drugs will also increase the risk of bleeding. Vascular anatomy (17) shows that the branches of the middle knee artery are distributed in the posterior condylar recess, the posterior half of the medial and lateral ventricles of the knee joint, and the posterior horn of the medial meniscus, forming a vascular plexus extending into the intercondylar fossa and supplying the anterior and posterior cruciate ligaments. The arterioles of the medial and lateral knee arteries form a periteniscal capillary plexus and annular vascular network in the synovium and joint capsule, and the meniscus; the lateral knee artery surrounds the lateral tibial condyle anteriorly and anteriorly from outside, immediately adjacent to the edge of the lateral meniscus body; the inferior transverse branch of the peripatellar artery network runs in the infrapatellar fat pad. These vessels, which supply the anterior and posterior cruciate ligaments, menisci, synovium, joint capsule, and fat pad, carry a high risk of injury during arthroscopic knee surgery, opening the possibility of hidden blood loss. In addition, the use of a tourniquet in patients also destroys the soft tissue at the extrusion site and leads to an increase of a certain amount of interstitial hematocele. Hemolysis, as another factor in the mechanism of action of hidden blood loss, is often easily overlooked. The use of a tourniquet effectively reduces significant blood loss seen intraoperatively, but excessive tourniquet time and reperfusion of limb blood loss after loosening can present additional injuries. Reactive hyperemia of the limbs can lead to a release of the tissue plasminogen activator from the vascular endothelium, which increases the fibrinolytic activity and accelerates the hemolytic reaction, resulting in hidden blood loss after surgery. After prolonged ischemia of tissues, there is a compensatory increase of anaerobic metabolism, activating a large number of oxygen-free radicals, and the body is in a state of oxidative stress (18), which can lead to red blood cell injury and induce hemolysis in the body (19). Hemolysis can further activate oxidative stress (20), resulting in hidden blood loss (21).

Prolonged surgery and blood transfusion also induce hemolysis, which is associated with the use of cardiopulmonary bypass (22) and also with oxidative cell damage (23). Transfused patients experience elevated bilirubin and decreased hemoglobin increment (24), which indicates that hemolysis is also present after transfusion, and it can also lead to hidden blood loss. In addition, gastrointestinal bleeding due to stress ulcers is also responsible for the increase in occult bleeding volume. Patient studied in the literature (25) was hospitalized several times for severe anemia of unknown origin (Hb 40-50 g/l) due to hidden blood loss from the jejunum, which was effectively controlled after treatment with gastrointestinal hemostasis. Perioperative patients are at risk of developing stress ulcers, which may lead to clinically important bleeding, and stress ulcer prevention should be taken seriously in patients in intensive care units and general wards (26).

There are many factors affecting hidden blood loss in the minimally invasive treatment of the knee joint. We hereby study to find out the related factors and guide clinical practice according to the mechanism affecting hidden blood loss. We selected 100 patients with knee joint injury who received arthroscopic minimally invasive treatment. According to the inclusion criteria, there were 58 males and 42 females, aged 17–66 years, with a mean of (44.78 ± 13.67) years; mean BMI (25.07 ± 3.67) kg/m^2^; mean total actual blood loss in the study population (512.32 ± 317.29) ml; and mean hidden blood loss (387.02 ± 252.56) ml. It can be seen from the characteristics of the study population that a major part of the population is middle-aged, mainly male, with hidden blood loss accounting for approximately 76% of the actual blood loss. It can be seen that hidden blood loss is predominant in a majority of the population, which is consistent with the findings of other minimally invasive surgeries (12). Gender differences associated with hidden blood loss were found in orthognathic surgery studies, with equal gender-related percentages of blood loss (27), which is consistent with our findings. However, in another study of perioperative hidden blood loss in patients undergoing posterior lumbar fusion, gender was not associated with hidden blood loss (28). Interestingly, among patients treated with anterior cervical discectomy and fusion, male patients were an independent risk factor for hidden blood loss (29). Hidden blood loss in females was found more in univariate analysis, which was consistent with the meta-analysis exploring that the risk factors of perioperative hidden blood loss in Chinese patients with femoral fracture were female patients (30), which may be related to the unique physiological structure of women. These discrepancies may be due to different study designs, surgery methods, and perioperative management. In laparoscopic cholecystectomy, hypertension is a contributing factor to hidden blood loss (31). There is a high hidden blood loss during spinal perioperative surgery, and studies have shown that hypertension, diabetes, and heart disease are all risk factors for hidden blood loss (32). Patients with medical diseases often undergo changes in hemodynamics and blood composition, and such patients have coagulation disorders, abnormal cardiopulmonary compensation, poor vascular elasticity, and lower tolerance to anemia, which all lead to more hidden blood loss (33). Most of the patients in this study with a long hospital stay were young patients with high-energy injuries and multiple knee injuries, which potentially increase the risk of occult bleeding.

Moreover, our study demonstrated that preoperative hematocrit, blood volume, and postoperative hematocrit were independent factors associated with actual blood loss, while preoperative hematocrit, blood volume, postoperative hematocrit, and gender were independent factors associated with hidden blood loss. Through a multifactor comprehensive analysis, considering that the muscle and bone content of male patients was significantly higher than that of female patients, these results determined that the male patients had higher preoperative blood volume and preoperative hematocrit. When knee joint injury and minimally invasive surgery occurred, it was accompanied by more muscle and soft tissue injury, especially when the bone tunnel was made, and it was accompanied by more bone loss and bone injury, resulting in more severe hidden blood loss, as demonstrated by lower postoperative hematocrit. According to the present study results, we recommend that in minimally invasive knee surgery, stable normal blood pressure be maintained to prevent more hidden blood loss induced by blood pressure fluctuations, and the tourniquet use time be controlled to prevent local soft tissue injury caused by tourniquet and ischemia-reperfusion to induce more hidden blood loss. Also, the following is recommended: radiofrequency ablation knife hemostasis after loosening the tourniquet to reduce hidden blood loss (34); appropriate perioperative administration of drugs to protect the gastric mucosa to prevent the occurrence of stress ulcers; appropriate use of antioxidants, such as vitamin E and carotene, to prevent hemolysis. It is suggested to use tranexamic acid to reduce blood loss in arthroscopic surgery (35). Studies have shown that (36) tranexamic acid, combined with systemic and local application, has important clinical significance in reducing perioperative blood loss and blood cell loss in patients with femoral fracture, with good safety; it is recommended to cold-compress the incision and surrounding tissues, as low temperatures can constrict capillaries and reduce local oxygen consumption, thereby reducing hidden blood loss (37).

There were some limitations in the current study. First, the sample size was relatively small. Second, this was a retrospective study and there was no control group. In addition, some variables such as the timing of drain removal and liquid balance, which may influence the outcome of hidden blood loss, were not included, and thus, these warrant further investigation. Last but not least, this was a single-center study, so the findings need to be subjected to further validation.

## Conclusion

Preoperative hematocrit, blood volume, postoperative hematocrit, and gender are independent factors for hidden blood loss in patients undergoing minimally invasive treatment of the knee joint. More attention to, and adequate management of, these factors will help reduce postoperative hidden blood loss.

## Data Availability

The raw data supporting the conclusions of this article will be made available by the authors, without undue reservation.
